# A global pilot comparative, cross-sectional study of clinical research nurses/research midwives: Definition, knowledge base, and communication skills related to the conduct of decentralized clinical trials

**DOI:** 10.1017/cts.2024.535

**Published:** 2024-05-06

**Authors:** Elizabeth A. Johnson, Gordon Hill, Hazel Ann Smith, Lisa Marsh, Kelly Beer

**Affiliations:** 1 Mark & Robyn Jones College of Nursing, Montana State University, Bozeman, MT, USA; 2 School of Health and Life Sciences, Glasgow Caledonian University, Glasgow, SD, UK; 3 School of Health, Science, and Wellbeing, Staffordshire University, Stoke on Trent, UK; 4 Buntain College of Nursing, Northwest University, Kirkland, WA, USA; 5 Centre for Molecular Medicine and Innovative Therapeutics, Murdoch University, Murdoch, WA, Australia

**Keywords:** Clinical research, clinical research nurse, communication, decentralized trial, research midwife, teletrial

## Abstract

**Background:**

A gap in the literature exists pertaining to a global research nurse/research midwife resources and communication skill set necessary to engage with participants of diverse populations and geographic regions in the community or home-based conduct of decentralized clinical trials.

**Aims:**

An embedded mixed methods study was conducted to examine research nurse/research midwife knowledge base, experiences, and communication skill sets pertaining to decentralized trials across global regions engaged in remote research: the USA, Republic of Ireland, United Kingdom, and Australia.

**Methods:**

An online survey was deployed across international research nurse/research midwife stakeholder groups, collecting demographics, decentralized trial experience, barriers and facilitators to optimal trial conduct, and the self-perceived communication competence (SPCC) and interpersonal communication competence (IPCC) instruments.

**Results:**

86 research nurses and research midwives completed the survey across all countries: The SPCC and IPCC results indicated increased clinical research experience significantly correlated with increased SPCC score (*p* < 0.05). Qualitative content analysis revealed five themes: (1) *Implications for Role*, (2) *Safety and Wellbeing*, (3) *Training and Education*, (4) *Implications for Participants,* and (5) *Barriers and Facilitators*.

**Conclusions:**

Common trends and observations across the global sample can inform decentralized trial resource allocation and policy pertaining to the research nurse/research midwife workforce. This study demonstrates shared cultural norms of research nursing and midwifery across varied regional clinical trial ecosystems.

## Introduction

Clinical trials are used to investigate the safety and efficacy of thousands of medications and devices every year [1]. Across the globe, clinical trials serve as the bedrock for medicinal advancement for a wide spectrum of diseases and indications, such as cancer and diabetes. Clinical trials are designed by sponsoring organizations, such as government entities or pharmaceutical companies to meet the rigor and data necessary to submit for commercial approval. Some common designs include the gold standard of randomized controlled trials (where people are randomly allocated to receive or not receive the intervention) and crossover trials, which can benefit expansion to participants on ineffective regimens (United States Food and Drug Administration [US FDA]) [2].

## Decentralized (remote) clinical trials

While nomenclature may vary, a decentralized clinical trial (DCT) is a model of clinical research that emphasizes the use of technology, direct shipment of investigational products, and mobile or local healthcare providers to conduct research-related procedures nearby or within a participant’s community or home environment [3,4]. Sometimes called remote or virtual clinical trials, the DCT model and hybrid trials (which have decentralized and traditional trial components in their design) have become popular among industry clinical trial sponsors as a means of adapting to the logistical challenges of participant recruitment, retention, and accessibility to trial opportunities.

The coronavirus disease 2019 (COVID-19) pandemic propelled the implementation of DCTs and expedited the use of remote monitoring capabilities, remote visits via telehealth, and the concept of community research engagement (mobile health units, bringing clinical trials to the patient) [5]. Since 2020, clinical trial starts have increased by 14% internationally, with over 6000 investigational drugs currently evaluated across all trial phases, with many utilizing either full or components of DCT structure [6]. In one retrospective study of 220 protocols, over 90% were classified as having decentralized trial model elements, with the most common being mobile applications and use of device technologies [7].

Internationally, almost 3 out of 4 persons enrolled in clinical research trials live 2 or more hours away from the research site, which inhibits completion of the average 12 in-person visits typical to a clinical trial [8]. The DCT design has the potential to improve access to novel therapeutics and treatments for underrepresented and underserved populations by removing barriers associated with trial locations and on-site appointment requirements. For many life-limiting conditions, such as cancer, clinical trials provide critical opportunities to access emerging treatments that may slow disease progression and prolong quality of life. Implementation of DCTs supports wider opportunity and accessibility to clinical trials, dissolving traditional barriers for populations excluded due to financial constraints, geographic location, and race/ethnicity [9]. Industry trial sponsors as well as national clinical research funding bodies are utilizing DCT design to enhance trial recruitment and enrollment.

## Role of the research nurse/research midwife with DCT conduct

The research nurse/research midwife role is a specialized, all-encompassing presence in a community as an advocate, healthcare professional, researcher, and liaison for participants to engage in research in a culturally aligned manner [10]. This expert knowledge related to assessments, participant visit schedule, and the investigative medicine/device is critical to the safety of the participant [11]. Additionally, as the DCT design enables participants to receive novel treatments via research studies/clinical trials at home, this expert knowledge also allows the research nurse/research midwife to accommodate for differences in care delivery environments (home instead of hospital or research center). The research nurse/research midwife is a skilled communicator, relaying information related to risks, benefits, and study schedules to participants and caregivers in a manner that is easily understood and applicable to the research-home setting [11].

There is a paucity of literature surrounding the specific communication skill sets required by research nurses/research midwives to align with community cultures, participants, and varied industry sponsors. Verbal and nonverbal communication skills have been linked to indicators of optimal clinical trial conduct, such as improved informed consent and accrual of participants [12]. However, specifics related to interpersonal communication skills as well as self-perception of communication skills in DCTs have not been described in the literature. Communication skill set directly relates to participant safety and trial data integrity and warrants exploration of this identified gap across research nurse and research midwife populations in the USA, UK, Republic of Ireland, and Australia (common regions for DCT deployment).

Current literature surrounding DCTs omits the description of research nurse/research midwife education requirements unique to DCT conduct compared to on-site research conduct and distinct to community-based clinical roles [13,14]. Without familiar equipment, resources, or surroundings, research nurses/research midwives require education relating to home-based delivery of clinical research, which requires synergy of their research-based training and nursing/midwifery training. For example, the dynamics with caregivers and the variability of resources available in the home or community practice milieu may necessitate an augmented approach to ensuring research data integrity and also with quality care, such as consistent Internet availability or sufficient physical space for procedure preparation. Clinical assessment and appraisal of a participant’s health status may be nuanced given the intersection of standard-of-care treatment and investigational product inclusion, which requires skillful communication and additional learning to distinguish evolving safety events possibly attributable to a study drug rather than a common side effect of a standard treatment [15]. Lack of education components surrounding the unique considerations of home or community-based research conduct with DCTs at the forefront has created a quality chasm for DCT participants in the arenas of safety, deviations from the protocol, data integrity, and cultural incongruence when in a participant’s home or local community [16].

## Purpose and research aims

While there is growing evidence of the importance of DCTs, there remains a gap in research nurses’ and midwives’ definition, knowledge base, and communication skills related to the conduct of DCTs. This study explores the remote/DCT approach within four countries that have adopted DCTs as a priority research design – Australia, the Republic of Ireland (RoI), United Kingdom (UK), and USA. The purpose of this study was to assess research midwives’/research nurses’ definition, knowledge, experiences, and communication skill sets related to the conduct of DCTs in an international context. The research aims were as follows:

### Aim 1

Explore the extent of research nurse/midwife exposure to DCTs through description of lived experiences, including participant management, safety, and their professional role, via a narrative response in an online survey.

### Aim 2

Measure research nurse/midwife self-perceived communication competence (SPCC) using the 12-item SPCC scale [17].

### Aim 3

Measure research nurse/midwife self-reported communication competence using the 30-item interpersonal communication competence (IPCC) scale [18].

## Guiding theory, conceptual model, and proposed adaptation for decentralized trials

This study is guided by an adaptation of the Nursing Role Effectiveness Model [19–21] and informed by communication accommodation theory (CAT) [22].

## Nursing Role Effectiveness Model

As described by Irvine and colleagues [19], the Nursing Role Effectiveness Model was created as a means of depicting the contributions of providers to patient and organizational outcomes within the context of the nurse’s role. These contextual factors, or components, are organized into three levels: structure, nurse role, and outcome. Structure relates to patient, nurse, and organizational contributions to the boundaries of a nurse’s role, such as staff mix, workload, nursing experience, and patient health status [19]. This model has been utilized in nursing research to evaluate the impact of nursing roles on patient outcomes and efficient care coordination among clinical providers [20,21].

## Communication accommodation theory

CAT is used to explore interpersonal communication and self-perceived effectiveness of communication through a multicultural lens. Communication is comprised of verbal and nonverbal behaviors as well as mental models (schemas, perceptions) that together influence the meaning of the interaction that facilitates communication and the meaning of the information relayed from one individual to another. CAT is selected as a guiding theoretical framework given its acknowledgment of sociohistorical context on interpersonal communication during participant-nurse interactions [22,23]. Given the DCT design is foundationally built upon virtual/remote communication, the core of the adapted model is communication accommodation, which is the nurse/midwife’s alignment to the changing communication conditions for each structural component and within the bounds of the nursing/midwife role.

## Methods

A mixed methods embedded design was employed comprising a quantitatively based online survey with the use of validated communication instruments. To enhance the trustworthiness and credibility of findings, methodological triangulation supported the credibility and dependability of this study via the SPCC and IPCC communication instruments as well as the narrative responses to barriers and facilitators of decentralized trial model deployment [24]. Confirmability was enhanced through consensus building and rounds of review among all researchers to arrive to non-biased agreement of findings interpretation of both qualitative coding-theme generation as well as the quantitative data analysis. Transferability was a key point of this study, as four separate regional samples of clinical research nurses and midwives were represented and compared [24]. Authenticity of the findings was strengthened with *in vivo* exemplars to increase the truth value of the themes, sub-themes, and coding methods employed [25]. In alignment with the research questions, STATA version 17 was utilized for descriptive statistics, variance analyses, logistic regression, correlation coefficients, and communication competence instrument subscale scoring analyses [26].

This study adhered to the data security policies of Montana State University, which included the utilization of the secure, encrypted Knox (data repository) account. Raw data output and general analysis documentation were stored within this Knox account, including team meeting presentations and compensation records. Only researchers and team members associated with the study had access to the Knox account, with no account permission sharing. The exchange of study materials occurred through a secure file transfer platform connected to the Knox account with encrypted links that had expiry dates associated to ensure timely access.

### Self-perceived communication competence (SPCC) scale

The SPCC is a measure of an individual’s self-perceived ability to convey information via verbal or nonverbal mediums of communication [17]. SPCC is a means of understanding how research nurses and research midwives self-evaluate their ability to communicate. The SPCC is a 12-item scale developed by McCroskey and McCroskey [17] that has been used in over 50 US and global studies [27]. The items in the SPCC prompt the participant to respond on a scale of 0 (completely incompetent) to 100 (competent) to statements such as “Present a talk to a group of strangers” and “Talk with a friend.” The SPCC has been cited with reliabilities ranging from 0.80 to 0.92; however, reliability and validity measures are dependent upon the context of communication within the specific culture and thus challenging to compare [27]. The utilization of SPCC for this study was exploratory in nature to evaluate its use to describe how communication competence is perceived in the research nurse/midwife population. This study will support the utility research of the SPCC within the cultural context of nursing communication.

### Interpersonal communication competence (IPCC) scale

IPCC centers on an individual’s ability to manage an interpersonal relationship when communication is involved [18]. Interpersonal communication is of paramount importance in nursing and midwifery to deliver culturally competent care within the dimensions of competence, such as empathy, supportiveness, and expressiveness [18]. The IPCC is a 30-item scale that has been utilized in nursing research and pedagogy as a reflexive tool, promoting critical thinking during complex encounters requiring communication skills [28]. Internal consistency of the IPCC is demonstrated by an overall Cronbach alpha of 0.86 [18]. The items within the IPCC prompt the participant to respond via Likert scale (from 1, almost never, to 5, almost always) to questions such as “Other people think that I understand them” and “I communicate with others as though they are equals.” Subscales for comparison include self-disclosure, empathy, social relaxation, assertiveness, altercentrism, interaction management, expressiveness, immediacy, and environmental control. The use of this instrument has been used in nursing and healthcare professional populations to evaluate communication as a critical factor to better patient outcomes [29]. This study utilized the IPCC instrument as an exploratory measure within the specific population of research nurses and research midwives to evaluate the ability and willingness to connect with clinical trial participants as a previously identified critical factor of the research nursing/midwife role [30].

### Qualitative data

The qualitative data were analyzed using Braun and Clarke’s method of thematic analysis [31]. Following this approach, initial codes were assigned to the data and organized in Microsoft Word and Excel by individual question and itemized participant responses by grouped region (i.e., US and outside US). *In vivo* coding was employed to enhance the truth value of theme creation as it retained the voice of the participant, keeping close the intended tone, meaning, and perspectives [25]. Themes were then established based on patterns observed among groupings of similar codes, reviewed for agreement among the researchers, and then summarily defined to ensure generalizable interpretation between US and outside US respondents. A consensus was reached with the research team after two rounds of agreement evaluation to increase the trustworthiness of the findings.

### Sampling and recruitment

Purposeful sampling was utilized for this study to garner the specific insights of research nurses and midwives in the regions of interest. The investigators met at consistent intervals via video conferencing to discuss recruitment, preliminary data trends, or findings, as well as updates on observations with ongoing news and publications surrounding decentralized/remote trial conduct. Snowball recruitment occurred from July 1, 2022, to September 29, 2022. Recruitment graphics and flyers which were approved by the Montana State University Institutional Review Board were electronically posted on Twitter, LinkedIn, and Facebook and shared via organizational email listings among the researchers. Recruitment partnership was established with the Irish Research Nurses and Midwives (IRNM), the International Association of Clinical Research Nurses (IACRN), and the Scottish Research Nurse, Midwife, and Coordinators’ Network. Email notices were sent to the members of IRNM and IACRN upon organizational approval, which included a brief overview of the study, investigator contact information by respective region, and a link to the survey. Within Australia, the survey was disseminated via informal CRN networks by email and through social media.

### Ethical considerations

This study was approved by the Montana State University Institutional Review Board in May of 2022 (Protocol #2022-193-EXEMPT). Participants were provided a consent overview prior to beginning the Qualtrics survey, which noted the right to withdraw from the study at any time. Participants had the right to not respond to questions on the online survey. To minimize psychological risks of discomfort with line of questioning, participant response to questions was voluntary, and questions could be skipped at any time. Lines of questioning reflected views and perceptions that would arise in the everyday life of the selected population (research nurses and research midwives). Privacy was maintained via secure, encrypted, and de-identified data collection within the Qualtrics survey and Knox data repository (Montana State University encrypted server). Email addresses provided by participants for compensation were only utilized for compensation; after the gift card was electronically delivered, the email address was destroyed. As this research was conducted online, there was no anticipation for research-related injury given minimal risk.

## Results

### Quantitative findings

A total of 86 eligible participants completed the Qualtrics survey. Of the 86, most respondents self-identified as residing in the UK (*n* = 40, 46.5%) followed by the USA (*n* = 30, 34.9%), the Republic of Ireland (*n* = 13, 15.1%), and Australia (*n* = 3, 3.5%). Demographic sample characteristics are displayed in Table 1. Participants identifying as residing in Australia are not isolated as a subgroup outside of the region-based demographic sample description in Table 1 due to the low total participation and potential ease of respondent recognition.

**Table 1.** Demographic characteristics of the sample population

Response item	*N*	Mean	Standard deviation	Range minimum	Range maximum
Age (y*ears*)	86	42.98	11.87	23	75
Gender					
*Female*	76 (*88.37%*)				
*Male*	9 (*10.47%*)				
*Nonbinary*	1 (*1.16%*)				
Region					
*United Kingdom*	40 (*46.51%*)				
*USA*	30 (*34.88%*)				
*Republic of Ireland*	13 (*15.12%*)				
*Australia*	3 (*3.49%*)				
Education					
*Associate*	5 (*5.95%*)				
*Bachelor*	43 (*51.19%*)				
*Master*	33 (*39.29%*)				
*Doctoral*	3 (*3.57%*)				
Professional role					
*Research nurse*	78 (*90.7%*)				
*Research midwife*	5 (*5.81%*)				
*Research nurse + midwife*	3 (*3.49%*)				
Years of experience *(total)*	86	16.94	11.82	1	55
Years of experience *(research)*	86	8.9	6.98	<1	34

Most of the sample who self-identified as a CRN (69%, *n* = 60) were relatively new to the research nursing, with 10 years or less of experience in supporting clinical research conduct (57%, *n* = 49), while 11.6% of CRNs (*n* = 10) noted as having between 11 and 20 years of experience. Research midwives comprised 5% of the sample. Of the population, those with 10 years of experience or less number *n* = 60 (69%), between 10 and 20 years’ experience number *n* = 20 (23%), and 20 years or more (*n* = 6, 7%).

The SPCC complete responses (*N* = 84) demonstrated a median score of 82.88 (out of possible 100) across all regions, with an interquartile range of 17.96. The average self-reported communication competence of complete responses was 79.03 (*SD* = 15.04; range 21.25–100). The sub-stratified SPCC subscales are noted in Table 2 for the overall sample.

**Table 2.** Self-perceived communication competence (SPCC) sub-stratification scores for overall sample

SPCC sub-stratification	*N*	Sample mean	Threshold for high SPCC	Threshold for low SPCC	Standard deviation	Minimum response score	Maximum response score
Public	85	75.75	86	51	17.6	21.7	100
Meeting	84	74.87	85	51	17.4	19.3	100
Group	85	80.77	90	61	17.9	17	100
Dyad	85	85.63	93	68	14.8	22.7	100
Acquaintance	85	79.93	92	62	15.4	26.5	100
Friend	85	86.44	99	76	13.9	20.8	100
Stranger	84	71.18	79	31	19.5	7.3	100

Across all sub-stratified levels and overall scores, no communication scenarios of basic communication contexts or receiver types reached scores indicating high SPCC [17]. The lowest SPCC average score was the “Stranger” receiver sub-stratification (71.18) and the lowest minimum participant response (7.3), while the highest SPCC average score was the “Friend” receiver sub-stratification (86.44) and highest minimum response score with the “Acquaintance” at 26.5.

The IPCC section of the survey totaled to 81 complete responses, with a median score of 3.77 out of a possible 5 (interquartile range of 0.7). The average IPCC score (*N* = 81) was 3.62 (*SD* = 0.56; response range 2.07–4.43). The summative findings for IPCC scores within the 10 domains of communication characteristics and frequency of associated behaviors are noted in Table 3.

**Table 3.** Interprofessional communication competence (IPCC) score summaries by domain

IPCC domain	*N*	Mean	Median	First quartile	Third quartile	Interquartile range
Self-disclosure	85	3.72	3.67	3.33	4.33	1.0
Empathy	84	3.64	4.0	3.0	4.33	1.33
Social relaxation	84	3.55	4.0	3.0	4.33	1.33
Assertiveness	85	3.65	4.0	3.33	4.0	0.67
Altercentrism	85	3.53	3.67	3.0	4.0	1.0
**Interaction management** ^*^	**85**	**4.05**	**4.33**	**3.67**	**4.67**	**1.0**
Expressiveness	85	3.78	4.0	3.33	4.33	1.0
Supportiveness	84	3.52	3.67	2.33	4.67	2.33
Immediacy	84	2.97	3.0	2.0	4.0	2.0
Environmental control	84	3.84	4.0	3.67	4.33	0.67

* Denotes highest mean and median score across total sample population.

The highest mean and median score across all regions was with interaction management, indicating that the respondents in this sample communicated *often with smooth shifts from one topic to the next* during conversations and took charge of conversations by *negotiating the topic of the conversation* and perceptiveness pertaining to *what people say but also what they do not* [18].

From the data set, hypotheses were drawn regarding potential variables affecting the SPCC and IPCC scores. Such potential variables included job title, years of experience (overall experience as well as research-specific experience), region, age, and gender. Partial and semi-partial Pearson’s correlations were generated, including Cramer’s V for categorical variables, as well as regression analyses of these variables. No identified variables were determined to have a statistically significant correlation with or effect on the IPCC score. Some variables indicated a positive correlation with SPCC scores and were investigated further. Of the identified variables, only one resulted in a statistically significant (*p* < 0.05) relationship to SPCC, which was “Years of Experience in Research.” Similar and related variables were tested and ruled out as being not statistically significant – “Years of Experience” and “Job Title” were not statistically significant indicators of SPCC score. The relationship between “Years of Experience in Research” and the resultant SPCC scores was a moderate, positive correlation and indicated that for each year in a research specialty, SPCC scores would increase by 0.55 points from an intercept of 74.32. It is also interesting to observe that the R-squared value of this analysis resulted in only 0.0683 – this is to say that while a statistically significant factor, this variable only accounts for about 7% of the natural variability of the SPCC score. Attempts to incorporate additional variables in the regression model of SPCC scores noticeably reduced the statistical significance of the model, did not result in any increase in the R-squared value, and did not diminish the relative statistical significance of the “Years of Experience in Research” variable within the model.

### Qualitative findings

Following the approach described in the methods section, the qualitative findings from this study identified five overarching themes: (1) *Implications for Role*, (2) *Safety and Wellbeing*, (3) *Training and Education*, (4) *Implications for Participants,* and (5) *Barriers and Facilitators*. These can be seen in Supplemental Table 1, alongside detailed information that helps to highlight the process that the researchers followed. These themes reflect the data that was received from the participants and highlight areas that were deemed to be positive and others that were perceived to be more challenging. Overall, the findings indicate a wide range of perspectives that may suggest the lived experience of conducting DCTs/remote trials is pervasive among the research nurse/research midwife group.

Supplemental Table 1 also details exemplars from the USA and a separate section for exemplars from the UK, RoI, and Australia, indicating some degree of accordance. However, it is noted that there was some contradictory data from the participants, for example, under the theme of safety. This indicates that the picture is complex and may be influenced by contextual factors that this research was not able to fully explore. The rationale for the geographical split of the exemplars was decided upon as the data from the UK and RoI were deemed to be similar and the data from the sample population from Australia was too small to be categorized on its own.

*Implications for Role* explored how DCTs/remote trials impact the role of the research nurse/research midwife. This theme includes the wide-ranging implications for practice, the scope of the research nurse/research midwife, delegated duties (and to whom), workload, and how the respective role is perceived. Collectively, these highlight how the implementation of DCTs/remote trials can significantly change the practice of research nurse/research midwife. The wide range of responses received indicates that this can have positive or negative ramifications. The theme of *Safety and Wellbeing*, which encapsulated both research nurse/research midwife and participant safety, was found to show divergence between US and non-US respondents. US respondents described safety concerns from the perspective of legal liability (licensure, medical, or decision-making errors) and hostility concerns in the home environment, which may propagate increased stress and anxiety due to the isolation and higher degree of skill set necessary to complete tasks alone. Non-US respondents perceived safety as a challenge in more of a virtual realm, seeing the benefits of less potential hostility during computer-delivered trial visits but also recognizing stressful communication challenges which may arise without face-to-face contact. *Training and Education* highlighted the need for a greater understanding of the implications of DCTs/remote trials, with many respondents identifying that they had received little, or no, training or education on these types of trials. This was apparent from all the contributing countries. The theme of *Implications for Participants* encapsulated many of the benefits of DCTs/remote trials for the participants as there was less need for travel to the hospital for trial-related procedures. Importantly, it was also highlighted that this could have a positive impact on the recruitment and retention of trial participants. However, some more negative comments related to some participants regretting that they could not have more face-to-face contact with the trial team. Lastly, *Barriers and Facilitators* identified that there were structural problems with DCTs/remote trials, including lack of hardware, access to information technology (IT) packages, specifically firewall issues in the UK National Health Service, and general Internet access issues. Problems related to the training of participants were also highlighted as a potential barrier. Suggested facilitators included standardization between studies, engagement with hospital IT groups, involvement of nurses/midwives at an early stage in an advisory capacity, and training.

## Discussion

Synergistic interpretation of the quantitative and qualitative findings suggests that while communication is a significant factor in the conduct of decentralized clinical trials from the research nurse/research midwife perspectives, there are still unknown additional contributors to our understanding of interpersonal and self-reported communication skill set or behaviors in a real-world setting. The significance found in the years of total research experience demonstrates the importance of specialty-related expertise and training associated with clinical research. These statistical findings are reflected in the qualitative exemplars (Supplemental Table 1) where respondents across regions described communication across multiple thematic groups were contextualized by the question-based scenario (e.g., communication as it relates to participant management or communication as it pertains to generalized safety). Respondents further described their willingness and desire to expand their training and education related to decentralized trials, demonstrating the commitment of the specialty to expert practice. Specialized training for the DCT role would have an applicability across the research sector, with an increasingly diverse range of professional research delivery roles alongside DCT growth. International research nursing organizations, such as IACRN, have advocated for nursing and midwifery voice in the standardization of guidance with the US FDA to heighten prioritization for awareness of the research nurse/midwife role and necessary resources for DCT conduct [32].

### Limitations

There are limitations to this research within both design and methodology. Limitations attributed to the study design include the cross-sectional collection of participant responses. As these participants are not followed longitudinally, this research is dependent upon participant accuracy. The lack of consistent administration timing of the survey across all participants may cause variance in response truth value as mood and attitude will change throughout the day. While the online survey permits global participation, there is always a potential for an increase in missing data due to a lack of participant response as well as risks of robot-derived false data (“bots”). There are noted challenges with utilizing the SPCC and IPCC given potential differences in participant conceptualization of communication through the lens of their schema, lived experience, and culture. To account for these limitations, the research team utilized a secure research platform, Qualtrics, to reduce bot responses. The survey also included grand-tour questions that permitted participant expansion of thought, which increased information power should a participant choose not to respond to all questions and variance within responses. The research team also incorporated a mobile device-friendly viewing version of the survey within the Qualtrics platform to address readability/visibility issues on smaller digital screens.

The research team adopted an international approach to recruitment to strengthen the global applicability of findings; however, the low participation numbers from Australia limit the generalization of findings within that region. Within Australia, unlike the US and UK/RoI, there is no national research nurse/research midwife network established. Subsequently, there was reliance on informal networks and social media engagement for recruitment for this survey. While a strong driver of engagement with the survey was likely the experience of research nurses/research midwives with DCT approaches during the COVID-19 pandemic, the Australian research nurse/research midwife experience was significantly different due to the comparatively limited impact of COVID-19 on clinical trial activity within Australia. Research nurses/research midwives within Australia may not have yet been exposed to the DCT model or decentralized trial elements; however, a nationwide model for teletrials (remote trials) has been successful in its implementation [33,34]

## Conclusion

There was considerable consensus across each region related to barriers and facilitators to optimal remote, virtual, and decentralized trial conduct and the professional role of the research nurse or research midwife in this evolving model of trial delivery. As more regulatory and government groups turn attention to generating guidance and best practices, research nurses and midwives are key stakeholders to bring voice to operational resources necessary to bring trial access to populations otherwise disadvantaged due to geographic location, travel burden, or other constraints [35]. As the profession of nursing diversifies and expands in the clinical research specialty, there is a global call to integrate additional training, education, and awareness pertaining to decentralized/remote trial models among healthcare systems, places of nursing education, and professional organizations providing continuing education for research nurses and research midwives. By supporting specialized nurses and midwives aiding in the conduct of clinical research in local communities, the promise of opportunity equity for research participation can be realized.

## Supporting information

Johnson et al. supplementary material Johnson et al. supplementary material

## Supplementary material

The supplementary material for this article can be found at https://doi.org/10.1017/cts.2024.535.

## References

[1] Park JJ , Thorlund K , Mills EJ. Critical concepts in adaptive clinical trials. Clin Epidemiol. 2018;10:343–351. doi: 10.2147/CLEP.S156708. 29606891 PMC5868584

[2] United States Food & Drug Administration. Clinical trials: What patients need to know. 2018, January 4. Available at: https://www.fda.gov/patients/clinical-trials-what-patients-need-know. Accessed December 27, 2023.

[3] Santa-Ana-Tellez Y , Lagerwaard B , de Jong AJ , et al. Decentralised, patient-centric, site-less, virtual, and digital clinical trials? From confusion to consensus. Drug Discov Today. 2023;28(4):103520. doi: 10.1016/j.drudis.2023.103520. 36754144

[4] United States Food & Drug Administration. Guidance document: Decentralized clinical trials for drugs, biological products, and devices. 2023, May 1. Available at: https://www.fda.gov/regulatory-information/search-fda-guidance-documents/decentralized-clinical-trials-drugs-biological-products-and-devices. Accessed December 27, 2023.

[5] Flaherty KT , Doroshow JH , Galbraith S , et al. Rethinking cancer clinical trial conduct induced by COVID-19: an academic center, industry, government, and regulatory agency perspective. Cancer Discov. 2021;11(8):1881–1885. doi: 10.1158/2159-8290.CD-21-0850. 34290074 PMC8340848

[6] IQVIA Institute. Global trends in R&D 2022. Available at: https://www.iqvia.com/insights/the-iqvia-institute/reports-and-publications/reports/global-trends-in-r-and-d-2022. Accessed August 26, 2022.

[7] DiMasi JA , Smith Z , Oakley-Girvan I , et al. Assessing the financial value of decentralized clinical trials. Ther Innov Regul Sci. 2022;57(2):209–219. doi: 10.1007/s43441-022-00454-5. 36104654 PMC9473466

[8] Thakur S , Lahiry S. Digital clinical trial: a new norm in clinical research. Perspect Clin Res. 2021;12(4):184. doi: 10.4103/picr.PICR_278_20. 34760644 PMC8525789

[9] Goodson N , Wicks P , Morgan J , Hashem L , Callinan S , Reites J. Opportunities and counterintuitive challenges for decentralized clinical trials to broaden participant inclusion. NPJ Digit Med. 2022;5(1):1–6. doi: 10.1038/s41746-022-00603-y. 35513479 PMC9072305

[10] Godskesen TE, Petri S , Eriksson S , et al. When nursing care and clinical trials coincide: a qualitative study of the views of nordic oncology and hematology nurses on ethical work challenges. J Empirical Res Hum Res Ethics. 2018;13(5):475–485. doi: 10.1177/1556264618783555. 29998780

[11] Ustianowski A, Harman T. How the NIHR mobilised and adapted the UK research landscape to deliver COVID-19 studies. National Institute of Health and Care Research [NIHR]. Available at: https://www.nihr.ac.uk/blog/how-the-nihr-mobilised-and-adapted-the-uk-research-landscape-to-deliver-covid-19-studies/30611. Accessed December 27, 2023.

[12] Morgan SE , Finn A , Raley J , et al. Assessing communication practice during clinical trial recruitment and consent: the clinical trial communication inventory (CTCI), Clinical Trials in Vulnerable Populations. London, UK: InTech; 2018.

[13] Masoli JA , Down K , Nestor G , et al. A report from the NIHR UK working group on remote trial delivery for the COVID-19 pandemic and beyond. Trials. 2021;22(1):911. doi: 10.1186/s13063-021-05880-8. 34895305 PMC8665850

[14] Johnson E , Marsh L. Clinical research nurse utilisation and role in the conduct of decentralised clinical trials: a literature review. J Res Nurs. 2023;28(3):214–226. doi: 10.1177/17449871231162497. 37332317 PMC10272696

[15] Miller TP , Marx MZ , Henchen C , et al. Challenges and barriers to adverse event reporting in clinical trials: a children’s oncology group report. J Patient Saf. 2022;18(3):e672–e679. doi: 10.1097/PTS.0000000000000911. 34570002 PMC8940729

[16] Tinkler L , Robertson S , Tod A. Multi-professional perceptions of clinical research delivery and the clinical research nurse role: a realist review. J Res Nurs. 2022;27(1-2):9–29. doi: 10.1177/17449871211068017. 35392190 PMC8980584

[17] McCroskey JC , McCroskey LL. Self-report as an approach to measuring communication competence. Commun Res Rep. 1988;5(2):108–113.

[18] Rubin RB , Martin MM. Development of a measure of interpersonal communication competence. Commun Res Rep. 1994;11(1):33–44.

[19] Irvine D , Sidani S , Hall LM. Finding value in nursing care: a framework for quality improvement and clinical evaluation. Nurs Econ. 1998;16(3):110–131. 9748972

[20] Doran DI , Sidani S , Keatings M , et al. An empirical test of the nursing role effectiveness model. J Adv Nurs. 2002;38(1):29–39. 11895528 10.1046/j.1365-2648.2002.02143.x

[21] Endacott R , Eliott S , Chaboyer W. An integrative review and meta-synthesis of the scope and impact of intensive care liaison and outreach services. J Clin Nurs. 2009;18(23):3225–3236. 19735339 10.1111/j.1365-2702.2009.02914.x

[22] Giles H , Ogay T. Communication accommodation theory. In: Whaley BB , Samter W , eds. Explaining Communication: Contemporary Theories and Exemplars. Mahwah, NJ: Lawrence Erlbaum Associates Publishers; 2007:293–310.

[23] Farzadnia S , Giles H. Patient-provider interaction: a communication accommodation theory perspective. Int J Soc Cult Lang. 2015;3(2):17–34.

[24] Lincoln YS , Guba EG. Naturalistic Inquiry. Thousand Oaks, CA: Sage Publications; 1985.

[25] Manning J. In vivo coding, The International Encyclopedia of Communication Research Methods. Hoboken, NJ: Wiley Online Library; 2017:1–2.

[26] Stata Statistical Software [Computer Software]. 2021. Available at: https://www.stata.com. Accessed December 27, 2023.

[27] Croucher SM , Kelly S , Rahmani D , et al. A multi-national validity analysis of the self-perceived communication competence scale. J Int Intercult Commun. 2020;13(1):1–12. doi: 10.1080/17513057.2019.1569250.

[28] Khalil IA , Hashish AE. Exploring how reflective practice training affects nurse interns’ critical thinking disposition and communication skills. Nurs Manag. 2022;29(5):20–26. doi: 10.7748/nm.2022.e2045. 35412033

[29] Ross JS. Randomized clinical trials and observational studies are more often alike than unlike. JAMA Intern Med. 2014;174(10):1557. doi: 10.1001/jamainternmed.2014.3366. 25111371

[30] Jones CT , Griffith CA , Fisher CA , et al. Nurses in clinical trials: perceptions of impact on the research enterprise. J Res Nurs. 2022;27(1-2):50–65. doi: 10.1177/17449871211073757. 35392186 PMC8980586

[31] Braun V , Clarke V. Using thematic analysis in psychology. Qual Res Psychol. 2006;3(2):77–101.

[32] International Association of Clinical Research Nurses [IACRN]. Comment from International Association of Clinical Research Nurses (IACRN): Comment ID FDA-2022-D-2870-0016. Available at: https://www.regulations.gov/comment/FDA-2022-D-2870-0016. Accessed August 20, 2023.

[33] Collins IM , Burbury K , Underhill CR. Teletrials: implementation of a new paradigm for clinical trials. Med J Aust. 2020;213(6):263–265.e1. doi: 10.5694/mja2.50741. 32815176

[34] Sabesan S , Malica M , Gebbie C , et al. Implementation of the australasian teletrial model: translating ideas into action using implementation science frameworks. J Telemed Telecare. 2023;29(8):641–647. doi: 10.1177/1357633X211017805. 34233548

[35] de Jong AJ , van Rijssel TI , Zuidgeest MG , et al. Opportunities and challenges for decentralized clinical trials: European regulators’ perspective. Clin Pharmacol Ther. 2022;112(2):344–352. doi: 10.1002/cpt.2461. 35488483 PMC9540149

