# Supplementary material for: A global pilot comparative, cross-sectional study of clinical research nurses/research midwives: Definition, knowledge base, and communication skills related to the conduct of decentralized clinical trials
Source: J Clin Transl Sci. 2024 May 6;8(1):e90. doi: 10.1017/cts.2024.535 (PMC11148821; doi:10.1017/cts.2024.535)
Supplement: Johnson et al. supplementary material [file S2059866124005351sup001.docx]

**Supplementary Table 1.** Themes, Sub-Themes, and Definitions of Qualitative Findings

| **Themes** | **Sub-theme** | **Number of Developed Codes** | **Definition** | **Initial analysis** | **Outside US Exemplars** | **US Exemplars** |
| --- | --- | --- | --- | --- | --- | --- |
| **Implications for role** | **Impact of Remote /Decentralized Trial Delivery on Role** | 4 | Describing remote or decentralized trial delivery through the context of the individual’s role as a research nurse/research midwife | - Community and/or home-based approach - Adaptation of Communication Methods - Integration of Technology in Trial Conduct | *Decentralised clinical Trial and/or hybrid design with one or more face to face visits*  *DCT, Home Trials Support, Home Care Research*  *Decentralization of clinical trial operations where technology is used to communicate with study participants and collect data*  *Remote trial activity or telemedicine* | *Studies executed through telemedicine and mobile/local healthcare providers, using processes and technologies differing from the traditional clinical trial model*  *Not all interactions between the site and subjects or the site and sponsor/CRO are in person. Many or most of the interactions are via telephone or telehealth services. This includes remote consent processes, remote data collection, even home visits.*  *To me, decentralized clinical trials encompasses conducting the bulk of the visits with the subjects while they remain at home. The visit is conducted via the internet, phone and/or a visiting nurse.* |
|  | **Scope of Practice** | 10 | The anticipated or actual impact of decentralized/remote trial elements integrated into the role of the research nurse/research midwife | - Technology Issues - Safety Concerns - Concerns of De-skilling - Licensure - Training Required - Broadened Knowledge Necessary for Trial Conduct | *Negatively as it would reduce physical assessments*  *Additional responsibility to assess patient via phone rather than in person*  *A lot of my scope of practice has moved from clinical based to ethical, professional, leadership and professional development based*  *It would limit the ability to see patients in person where you can assess them face-to-face for safety events and take away the ability to perform physical clinical skills which impacts on nursing practice*  *Remote trials can increase scope of practice, for example our team remotely co-ordinates a larger team for a fully remote trial. Prior to covid this would not have been in our job role* | *There is some push for practicing beyond scope of practice due to lack of availability of Doctors or APP's, you have to push back and say no, which is for the safety of the patient as well as protects your license*  *We are checking our licensure to see where we are allowed to see subjects for study visits other than our clinic. For instance, subjects that are admitted to a LTAC [Long Term Assisted Care] but require IP accountability/dispensing or lab draws...rather than trying to arrange transport to our site, we want to see if we can go to them.*  *With the increase of virtual and home nursing visits, I must maintain and increase my skills and knowledge. This can be accomplished by attending seminars, hands-on practice when learning a new technique or skill and asking questions of those who have demonstrated that knowledge or skill.*  *Inclusive of technology knowledge and possible travel* |
|  | **Delegated Duties** | 6 | Alterations in volume and type of delegated duties associated with decentralized/remote clinical trial conduct | - Increased Delegation to Nurses and Non-Nurses - Participant Responsibilities | *Need to upskill in new methodologies including electronic platforms to manage e-consent, digital marketing, electronic data capture*  *Likely more duties could be taken by junior staff, staff without a nursing qualification if it is more information gathering rather than nursing skills. May find it difficult to teach students as lacing face to face opportunity, less skills potentially to teach students if on placement and all studies are remote*  *Likely to increase delegated duties*  *Duties have changed to remote based - interacting and supporting participants, assisting with study start-up, administering informed consent, managing the eTMF and responding to S(AE)s* | *Have shifted in certain areas, for example duties we did before someone else that is on site is handling them, and those of us off site is handling a job usually performed by the person on site/areas*  *Sometimes there are blurred lines for which member of the interdisciplinary take ownership of particular duties*  *Participants would need to do more self-monitoring and reporting unless much more sophisticated telehealth systems were put in place*  *An increase in delegated duties. I would think there are many more steps to oversee in the virtual clinical trial environment. Accountability for delivery of study drugs/materials to participants' homes, confirming adherence to study procedures whilst observing remotely etc.* |
|  | **Workload** | 5 | Augmentation of workload with apparent and non-apparent tasks or task management | - Technology Issues - Time Allocation - Expectations with a Hidden Workload | *Unseen/Unrecognised workload by colleagues*  *Workload changes with changes to trial design. We need to re-think how we work. Need to work harder building relationships with trial participants as cannot do this via a face-to-face meeting. This has the potential to affect recruitment and retention if not managed well*  *More time consuming, if having to screen and approach remotely, particularly if it relies on other remote colleagues*  *It can result in an increased workload- for example taking several attempts to get hold of a participant, expected to work on multiple remote trials on the same day rather than allocated time slots for face-to-face clinics where only one trial can be prioritised at a time*  *Could increase as virtual isn’t always quicker, more requirements and IT issues*  *Negotiate expectations and set reasonable timelines* | *Some of this is made easier. Being able to do a lot from my computer means time saving. however, since some of the clinical research systems are not user friendly for remote monitoring, in some cases it can increase the workload. For instance, needing to use FaceTime to show a monitor IP for accountability. It's hard to get a clear shot!*  *Could reduce some work and/or make it more efficient (less time spent scheduling space at clinical site, less time traveling between clinic/office/etc. But if there are visits in the home, could be less efficient (drive time)*  *Greatly increased because of the sheer number of electronic systems one needs to access to make this all work (at least 5 internally plus 7-10 PER study externally) biggest challenge is remembering all the passwords*  *Currently, the DCTs are staffed external to the clinic staff by independent contractors*  *May increase nursing time with patient due to education and instructional needs as well as catering to patient schedules vs patients catering to provider schedule* |
|  | **Overall Perception of the Role of the Research Nurse/Research Midwife in Remote Trial Conduct** | 19 | Generalized reflections pertaining to the decentralized/remote clinical trial model as it relates to the role of the research nurse/research midwife | - Resources - Training - Concerns of De-Skilling - Lack of Contact with Participants - Safety Concerns | *Not always the infrastructure available to cope with remote activities (i.e., redacting and photocopying)*  *Being available is key regardless of time zones*  *The bonding between participant and research nurse will be gradually lost*  *It is a very interesting area and as we move into a more digital age, these trials will become more common*  *More research tasks are sometimes delegated to non-clinical staff as less need to see participants face to face and use clinical skills* | *I really don't even know how to answer this question at the present time as I think it is all too new*  *Include more participants in studies*  *I mainly teach, but to me this would require an understanding of training principles because the CRN will need to train individuals about the study- especially if data are collected external to the centralized research site*  *We need to be able to talk to each other. Share joys, triumphs, and challenges. Nothing like that exists because DCT is so new*  *Concern about safety of staff and patients in DCTs* |
| **Safety and well-being** | **Safety** | 6 | The impact of remote trial delivery on the role of the research nurse/research midwife as it relates to their safety and the participant’s safety | - Safety Benefits During the Pandemic - Confidentiality Issues - Working Alone - Accuracy of Nursing Assessment and Participant Status Interpretation | *Certainly, concerns around breaching confidentiality and GDPR governance with increased use of internet and sharing patient identifiable information electronically*  *Potentially safer in some aspects. Less exposure to infection or hazard. Less direct exposure to behavioural issues sometimes experienced in face-to-face meetings such as aggression or verbal outbursts from participants face to face*  *I worry about confidentiality, and it can exclude patients from trials that do not have access to a smart phone/computer*  *There is a risk of error for some remote studies, communication and participant understanding pre-consent is more difficult to determine without the face-to-face element* | *Going to a participant's home could be a safety risk depending on who is at the home (hostile friends/family, dangerous pets, smoking, drugs)*  *Increase in liability in certain areas e.g., ensuring correct study med, correct dose, correct time etc. Also, if home visits occur, is the research nurse expected to go alone? If there has been a safety event or miscommunication, this could lead to a level of unpredictability in participant behavior which could raise safety concerns for the research nurse/midwife's immediate environment.*  *Concern for safety of staff serving patients in their homes or in the communities. Back up resources need to be available.*  *Going to other locations might open us up to risk to our licensing. Also, assessments done via telephone or telehealth may not be as accurate. we could miss something, putting our subjects and our licenses at risk*  *Need to ask more meaningful assessment questions* |
|  | **Wellbeing of the Research Nurse/Research Midwife** | 11 | The influence of adjusted work environments and trial processes on the personal wellbeing of the research nurse/research midwife | - Lack of Contact with Participants - Stressful for Some Nurses - More Flexibility with Remote Work - Concerns Regarding Consent - Feelings of Isolation - Enhanced Wellbeing | *Very stressful not being able to see the person whilst consenting, you can't truly tell whether have understood the information*  *Initially could be quite stressful to set up but better in the long term once everything has settled*  *For myself, a large part of the satisfaction of the job is contact with patients. I find the relationship developed between patient and research nurse valuable and rewarding for both CRN and participant. A move to remote patient contact would significantly decrease my job satisfaction and therefore my wellbeing*  *Some remote studies can be repetitive, seeing patients face to face is a key part of job satisfaction being a nurse. I think being able to maintain some face-to-face studies will be a key factor in job retention in the future if more and more trials decide on remote recruitment and delivery. Personally, I don’t get the same job satisfaction from a remote trial as I do face-to-face*  *I am in a much better head space than I was while working in a hospital. I am not as stressed or burnt out as I was.* | *Increased stress and anxiety due to increase in workload and also oversight.*  *Research in the home is isolating and can be lonely. You miss the nurses' station banter and learning from your colleagues*  *We need a variety of computer-based skills, especially in the use of word processing, spreadsheets, database and presentation software, and the ability to undertake internet searches*  *May support wellbeing if only being remote; stressful though to avoid direct contact and ability to monitor in person may be stressful to have to travel and engage in an unfamiliar location*  *Lack of social stimulation, increased isolation* |
| Training and Education | **Educational preparation** | 24 | Training and/or education the research nurse/research midwife has received or would request to receive related to decentralized/remote clinical trials | - Learning from Others - Training on Unique/Particular Topics Related to DCTs | *Not received any training. I would like to work alongside nurse who regularly use remote trials*  *I haven't received any training for remote trial delivery. The training needed will be depending upon the procedures to be performed to trial visit*  *How to correctly handle confidential information and how this is shared to those outside the NHS if sponsor or study using workers out with the NHS, how do we transfer information correctly via email etc. and what standards are in pace to protect participant information. Information on how to approach this with study sponsors to ensure the correct legal thing is being done. Difficult when working with academic studies or when studies required personal information to be shared. Is there a set SOP to use between all different type of studies i.e., commercial and academic to ensure the correct safe havens and databases are used so staff know at a glance during study set up if the sponsor has set up these communication systems correctly.*  *Have not received any training - I would like to standards and scope of practice established first. this will inform a competency framework from where we can develop training packages* | *My entire research career has been decentralized. I started in 2020, so I learned with the sponsors how to conduct a decentralized study*  *Learning about legal issues would be top interest*  *I am creating research documentation training for the DCT nurses. The combo of home care and research is new and still in its fledgling stages. Basic research knowledge is what is needed*  *How to practice safely; would need very specific SOPs prior to initiation*  *My particular institution has mandated the attendance of multiple classes, in-services on remote consent, telehealth, documentation of conversation with patient prior to their visit, during their visit, as well as following up on the co-signing of notes* |
| **Implications for participants** | **Participant Feedback** | 10 | Participant feedback as described and recalled by the research nurse/research midwife pertaining to the experience of actively engaging in a decentralized/remote clinical trial or study | - Generally Positive Feedback from Participants - More Flexibility - Benefits During Pandemic - Training Required for Participants - Potential for Disengagement | *Participants vary; if they keep up with participation when completed remote. We have one study that starts face to face them goes remote, there are a lot of people at disengage when they do not have a set appointment to attend therefore missing a lot of data and potentially needing to increase recruitment figures to account of those drop off rates*  *Less need to travel to sites for appointments, easier to take part, less time commitment overall*  *Difficult to pursue*  *Often glitches with IT, haven't received links or attachments or emails. Patients are mostly understanding and receptive of remote trials, particularly for covid where they recognise the need*  *Enjoy flexibility and not having to travel for appointments* | *They wish they could meet us and thank us in person. they are grateful we have continued the trials and found a way to work around COVID*  *Most enjoy the technology. Some would prefer more in-person visits and some fewer*  *They have stated that the virtual meeting was something that they hadn't done before and the technology was knew to them, but that they liked the ability to meet with the CRC at their home*  *They have enjoyed the flexibility* |
|  | **Participant Management** | 9 |  | - Reduced Participant Travel - Communication - Expansion of Recruitment - Remote Tasks | *Less reimbursements and travelling fees to arrange, but shorter follow up times beyond what protocol states to provide reminders for questionnaires or blood work. lots of calls to facilities and faxing i.e., blood forms to coordinate rural care. we also see quite a few missed study visits as patients are harder to reach to be reminded of upcoming visits.*  *Need to make sure that participant is competent and compliant - especially if taking medication at home and unable to check accountability*  *I feel there is even more support available to participants as they can email, phone or book a video call with a member of the clinical team to ask for support*  *It's sometimes hard to answer questions/problems that aren't asked. For example, if recruiting locally I can meet face to face and ask a direct question, resolving any eligibility criteria questions. But remotely I can see issues, but until the team ask for assistance it can be hard to approach for purposes of sensitivity*  *Greater use of electronic methods of communication* | *More telephone phone calls to follow up with patients in regards to diaries, symptoms, shipment of medication to participants "because they forgot to go to the pharmacy"*  *More visits are virtual, but many studies could collect information more efficiently with the use of an app or emailed questionnaire. I have spent so much time trying to get in touch with someone just to ask a simple questionnaire like "Do you have a cough?" "Do you feel tired?"*  *The ability to have a young family not travel to the site or an elderly dementia patient's loved ones that research can be done in the home has made tough complex trials easier to do*  *Requires a lot more follow up for return of study documents, a lot more coordination and financial resources*  *Implementation of consistent follow-up and tracking systems and practices* |
| **Barriers and facilitators** | **Barriers to Adopting Decentralized/Remote Clinical Trial Models** | 19 | Perceived barriers to the regional adoption of decentralized/remote clinical trial model | - Technology Supplies - Network Connectivity - Staffing and Contracted Services - Population Resources - Absence of Clinical Research Nurse/Research Midwife Input - Oversight and Regulatory Governance - Communication | *Internet connection is patchy and unreliable. The NHS firewall prevents access to numerous study online resources and special permission must be sought from a reluctant NHS security team - a time consuming activity. Layers of complex governance and protocols, written by various departments, around remote delivery must be navigated - again, challenging and time consuming*  *Large geographical area. Not all patients have access to mobile phones and internet*  *Research ethics and governance, funding models, lack of interest from our public hospitals in clinical research*  *Equipment. Real estate. Lack of foresight from sponsors by not including these measures*  *Barrier poor internet access for rural participants* | *The largest challenge is to ensure that the research staff understand their duties and more communication via phone and WebEx with the subject*  *In my current role I have no voice in study design for outside sponsored studies. Our organization also makes it hard to start up studies quickly*  *Certain age groups do not handle technology well. Some indigenous populations do not have access to technology or are at risk of having any tech stolen.*  *Using sponsor-provided home health services to conduct subject visits is HELL. They are on your DOA, but you can't necessarily see all their training, you don't get to supervise them or train them, you just have to accept what they do. And then you have to write the PDs for when they mess up. You're responsible for them, but you can't oversee them.*  *Urban/rural distances would be a barrier* |
|  | **Facilitators to Adopting Decentralized/Remote Clinical Trial Models** | 14 | Perceived facilitators to the regional adoption of decentralized/remote clinical trial model | - Expansion of Technology Support - Training and Cross-Training - Funding - Advocacy for Nursing Voice in Trial Design and Conduct - Investigational Product Transport - Standardized Policies and Procedures | *Training and standard policies to allow for consistency between sites. e.g., everyone follows the same GCP principals so needs to be standardised*  *Proper set up of electronic devices. To have access to NHS laptops, use of electronic signatures an IT department that understand research demands are different to general NHS hospital therefore there is a lot of back and forth of getting firewalls removed to allow specific study databases/equipment to be used*  *Appropriately trained and experienced nurses to establish and grow remote clinical trials - however government funding will need to also be secured*  *Better IT equipment*  *Increased funding to account for travel time and increased training* | *Money and staffing; questioning why decentralized would be discussed in the poor ability to keep and support current staffing levels in a controlled setting*  *More voice in study design before we agree to carry out the study. Sponsors just keep adding administrative burden without thinking through the impact on study teams*  *In-services for the home visiting research nurses to ensure thorough understanding of the protocols and research process*  *There must be assurance of drug stability and appropriate storage facilities in the patient’s home* |
